# Health Care Utilization by Adolescent/Young Adult Patients With Sickle Cell Disease in Delaware

**DOI:** 10.7759/cureus.22700

**Published:** 2022-02-28

**Authors:** Stephanie Guarino, Charmaine Wright, Sophie Lanzkron

**Affiliations:** 1 Hematology, ChristianaCare, Wilmington, USA; 2 Pediatric Oncology, Nemours Children's Health System, Wilmington, USA; 3 Center for Special Health Care Needs, ChristianaCare, Wilmington, USA; 4 Hematology, The Johns Hopkins University School of Medicine, Baltimore, USA

**Keywords:** population based study, adolescent young adult populations, health services utilization, pediatric to adult transition, sickle cell disease (scd)

## Abstract

Patients with sickle cell disease transition from the pediatric to the adult health care system during a vulnerable time in their lives, resulting in increased morbidity and mortality during this adolescent/young adult (AYA) period. The purpose of this study is to examine the health care utilization of a cohort of adolescent/young adult patients with sickle cell disease in the main adult health care system in Delaware. Analysis of an electronic health record (EHR) data set of emergency department encounters and inpatient admissions for all patients with sickle cell disease between July 2016 and June 2017 was performed. This revealed significant variability in health care utilization by adolescent/young adult patients with sickle cell disease. There was a small cohort of high utilizer patients with multiple emergency department visits and inpatient admissions. These high-utilizing patients might benefit from targeted interventions and a primary care medical home. By analyzing health care utilization and identifying the needs of this chronic disease cohort, a comprehensive care program specifically for adolescents/young adults could be developed to address the needs of the patients and to correct the gaps in the current system.

## Introduction

Sickle cell disease, an autosomal recessive hemoglobinopathy causing anemia, affects approximately 100,000 people in the United States according to the Centers for Disease Control and Prevention and is found predominantly in African-Americans [1]. Individuals with sickle cell disease experience a spectrum of chronic complications, including retinopathy, cerebrovascular disease, nephropathy, and pulmonary hypertension that persist across the life span. Although significant progress has been made in improving mortality for pediatric patients, the same gains have not been seen in adolescent and young adult patients, ages 15-39. Between 1999 and 2002, compared to 1995-1998, the mortality for pediatric patients aged birth to three years improved by 42%, whereas the mortality for patients 10-14 years remained stable, with no improvement [2]. Similarly, mortality rates have improved between 1979 and 2009 for pediatric patients aged from birth to 14 years, while they have not improved for patients aged 19 to 34 years [3,4]. 

The process of transitioning from pediatric to adult health care systems can be disjointed, contributing to the high morbidity and mortality seen in this population [5]. Research shows that patients with Type I diabetes experience poorer disease control and health outcomes during the transition period, underscoring the importance of appropriate monitoring and treatment during this time [6]. Previous research has shown that adolescent/young adult patients with sickle cell disease have higher emergency department utilization and lower primary care usage, highlighting the system failures that exacerbate the chronic condition of sickle cell disease [7].

Adolescent/young adult patients often access care at both pediatric and adult health care facilities resulting in fractured and incomplete medical care. Many patients also have complicated social and mental health care needs that would benefit from additional supportive services not typically part of an adult primary care office. These additional services often include behavioral health, case management, social work, and pharmacy services. While previous research has looked at specific factors that might help ease the transition process, less is known about how to create a comprehensive care program to care for patients with sickle cell disease in the adolescent/young adult age groups, especially one that spans multiple health systems [8].

Significant research exists around pediatric sickle cell care and the way these patients access to care. One notable study analyzed parent-reported data to assess the association between health care utilization, including Emergency Department (ED) visits and hospitalizations and outpatient care at a patient-centered medical home (PCMH). The authors specifically examined relationships between utilization and specific PCMH components and found that comprehensive care was associated with fewer ED visits and hospitalizations. The study included pediatric patients only and relied on parent self-report, which may be a source of error [9]. Similarly, adolescent/young adult patients with sickle cell disease cared for in a coordinated health care system in the UK experienced a mortality benefit. However, these results may be difficult to generalize, considering the key differences between the health systems in the UK and the United States [10].

Far less research exists on the health care utilization patterns for adolescent/young adult patients with sickle cell disease. Using five state Medicaid databases, the health care utilization patterns, specifically inpatient and emergency department utilization, of an adolescent/young adult cohort of patients with sickle cell disease were analyzed. This study found that the rates of emergency department reliance increased during the transition from pediatric to adult care, with the highest rates among adolescents/young adults. They also found higher rates of hospitalization and higher costs of care in adolescent/young adults than in pediatric patients. The study relied on administrative Medicaid claims data, which may be of questionable reliability since the data depend on the coding accuracy of the provider [11].

For adult patients, there are evidence-based guidelines for the care of patients with sickle cell disease in the United States; however, patients often do not receive the recommended care. Patients with sickle cell disease experience increased disease-related complications, especially when they do not have a primary care medical home, resulting in more ED visits and a higher burden of disease [12]. This research supports the need for patients with sickle cell disease to have a coordinated care home for better outcomes. In one study of sickle cell patients over 40 years old, only 38% had a primary care physician, and only 11% of eligible patients had a screening colonoscopy [13]. For those patients who have access to a primary care physician, evidence shows that these physicians lack confidence in treating patients with sickle cell disease and often rely on outdated information to make treatment decisions [14]. 

Little is known about how adolescent/young adult patients in Delaware access health care. Information about their health care utilization patterns could be used to inform the design of a state-wide comprehensive care program for patients with sickle cell disease within Delaware. This care center will need to span both the pediatric and adult health care systems in the state since patients in this age group access care across both health systems and through a variety of providers.

The goal of our project was to examine health care utilization, more specifically hospital admissions and ED visits, by patients with sickle cell disease in the main adult health care system in Delaware. The development of a comprehensive care center will benefit from the better understanding of health care utilization this project will provide. 

## Materials and methods

We conducted a retrospective analysis of all sickle cell disease patients who were admitted to one of the three EDs of Christiana Care Health System between July 1, 2016, and June 30, 2017. The study population was defined as patients who had an ED visit encounter with a primary or a secondary discharge diagnosis ICD-10code of D57.XX, which identifies sickle cell disease. We excluded patients with a coded diagnosis of sickle cell trait only. The encounters, which all began in the ED, included encounters that took place in the ED alone as well as those that ended with patients admitted to observation or inpatient units. 

A data set from Christiana Care’s electronic health record (EHR), PowerChart® (Cerner, Missouri, US), was obtained and comprised basic demographic information like age, sex, and zip code, as well as health care utilization data. This dataset included the number of ED visits, number of ED to hospital admissions, length of stay for both inpatient and ED admissions, as well as associated problems for each encounter. Further information on sickle cell genotype was not reliably available from the EHR. We did descriptive statistics to assess resource utilization, focusing on the adolescent/young adult cohort. This project was approved by the Christiana Care IRB by expedited review with a waiver of informed consent according to 45 CFR 46.116(d) and waiver of Health Insurance Portability and Accountability Act (HIPAA) Authorization according to 45 CFR 164.512(1)(i)2 (ii).

## Results

The dataset included 834 total emergency department encounters for 322 unique patients aged 18 to 92 years. Among this cohort, there were 149 unique patients aged 18 to 39 years with 343 emergency department encounters. Adolescent/young adult patients accounted for 46.2% of the total unique patients and 62.4% of the total encounters in the dataset. Male patients accounted for 51.6% of the adolescent/young adult encounters, whereas female patients for 48.4%. Of note, the Christiana ED, located in Newark, Delaware (DE), is the main ED for the health system with the highest overall patient volume, while the Wilmington Hospital ED, located in the urban downtown of Wilmington, has a much smaller overall volume. Yet, there was almost an even number of encounters at each of these EDs, 44.3% at Wilmington vs. 47.2% at Christiana. Additional demographic characteristics of the cohort are presented in Table 1. As shown on the map (Appendix 1), a large majority of the patients who had multiple ED and hospital visits were located in Northern Delaware.

**Table 1:** Encounter Characteristics

	AYA Cohort (%)	Older Patients (%)	All Patients (%)
Number of Encounters	343	207	550
Number of Patients	149	173	322
Age			
18-29	213 (62.1)	--	213 (38.7)
30-39	130 (37.9)	--	130 (23.6)
40-49	--	66 (31.9)	66 (12)
50-59	--	106 (51.2)	106 (19.3)
60+	--	35 (16.9)	35 (6.4)
Sex			
Male	177 (51.6)	133 (64.3)	310 (56.4)
Female	166 (48.4)	74 (35.7)	240 (43.6)
Encounter Type			
ED only	219 (63.8)	160 (77.3)	379 (68.9)
Observation Admit	20 (5.8)	10 (4.8)	30 (5.5)
Inpatient Admit	104 (30.3)	37 (17.9)	141 (25.6)
Encounter Location			
Christiana	162 (47.2)	65 (31.4)	227 (41.2)
Wilmington	152 (44.3)	137 (66.2)	289 (52.5)
Middletown	29 (8.5)	5 (2.4)	34 (6.2)

There was a large range of several inpatient and ED visits within the adolescent/young adult cohort. A total of 61 patients (40.9%) had only one ED visit or inpatient admission, while 10 (6.7%) patients had more than 10 ED encounters or admissions; this high utilization ranged from 10 to 24 encounters. Of note, in the 40-92-year-old cohort, there was one patient with 43 encounters, representing 7.5% of the total number of encounters.

Of the 343 encounters for adolescent/young adult patients, 124 (36.2%) resulted in the patient’s admission to either observation or inpatient status. In the other 219 encounters (63.8%), the patient was discharged from the emergency department or left against medical advice. The ED to inpatient admission rate was slightly higher at Christiana (73 of 162 encounters, 45.1%) as compared to the inpatient admission rate at Wilmington Hospital (47 of 152 encounters, 30.9%). All 29 encounters at the Middletown Emergency Department ended in discharge; there were no admissions. Of note, 29 encounters (8.5%) ended in the patient leaving against medical advice (AMA). Five patients (3.4%) had two or more AMA discharges; each of the three emergency department sites had at least one AMA discharge.

## Discussion

The analysis of this data set is the first attempt to understand the health care utilization of an adult cohort of patients in the state of Delaware, specifically adolescent/young adult patients. Delaware is unique as it has one main pediatric health system and one main adult health care system, with several smaller health systems in the southern part of the state. Thus, most patients seek care at one of these two institutions, especially those that live in New Castle County, the northernmost of Delaware’s three counties. New Castle County, where both the Christiana and the Wilmington EDs are located, is home to 58.5% of the state’s population. This helps explain the concentration of patients in these zip codes in the data set.

Furthermore, these findings confirm other geographic centers’ experiences, most notably that there are subsets of adolescent/young adult patients who are high utilizers of acute health care services like the ED. Previous research evaluated a cohort of adolescent/young adult patients with sickle cell disease over approximately 25 years at a single urban center. They found that a subset of the adolescent/young adult cohort had high utilization of ED, hospital inpatient, and outpatient clinic services, especially those with chronic pain, mental health comorbidities, or social and economic stressors [7]. Further analysis of our cohort and identification of the factors associated with higher utilization would allow more targeted interventions to minimize unnecessary health care utilization. The inpatient from ED admission rate of 38.2% from Christiana and Wilmington EDs is slightly less than Nemours’ pediatric data for sickle cell disease-related admission rates of 55% (unpublished departmental data). 

Analysis of the data set for the fiscal year 2016-2017 reveals there is a notable cohort of patients with sickle cell disease who access care in the Christiana Care Health System. Data were only obtained for one fiscal year as a preliminary set for analysis, and this small sample size is a limitation of the study. A larger project to track a cohort of adolescent/young adult patients with sickle cell disease during 10 years across health systems in Delaware was recently successfully funded as an extension of this initial project. This project is especially important to understand the health care utilization of adolescent/young adult patients as they transition between pediatric and adult health care systems. 

The data set only included acute care visits and inpatient admissions originating in the Emergency Department from one health care system in Delaware but is lacking outpatient care information. Obtaining this would allow a more complete understanding of overall health care utilization in this cohort as it would allow the study of patients with sickle cell disease who are healthy enough not to require acute care services. Another limitation is that this data set was created based solely on provider-coded billing diagnoses. Previous experience has demonstrated that this can be an inaccurate way to define a cohort of patients with sickle cell disease [15]. The data can be validated by further analyzing a random sample of encounters and reviewing specific clinical and laboratory documentation to confirm the diagnosis of sickle cell disease.

Additionally, there is a high utilizer population, some of whom had more than 20 acute care visits and admissions in these 12 months. This cohort may be target patients for the newly created Center for Special Health Care Needs Sickle Cell Program at Wilmington Hospital. This center includes primary care, hematology subspecialty care, behavioral health, social work, and case management and is described further below. In particular, one of the first goals of the Center is to explore the feasibility and impact of a day infusion center. Implementation at another large academic center decreased ED visit rates as well as readmission rates after hospital discharge. The infusion center was also associated with a significant improvement in the cost of care [16]. Future research should address the effect of this medical home on a high utilizing cohort of patients with sickle cell disease, with the expectation that these multidisciplinary services not only improve patient satisfaction and quality of life but also decrease the cost of care and increase the quality of care. The Comprehensive Care Program at the Center for Special Health Care Needs can serve as a model for the integrated care of other complex and chronic diseases beyond sickle cell disease.

## Conclusions

Sickle cell disease is a complex, chronic disease that has a spectrum of clinical manifestations in patients across the lifespan. This analysis, in conjunction with previously published research, indicates the need for focused interventions during the vulnerable adolescent/young adult period in patients’ lives. Often, patients face the complicated psychosocial stressors of adolescence and the development of identity and independence, which then are exacerbated by the chronic disease complications of sickle cell anemia. This interplay between physical health and the social determinants of health can lead to sicker patients who access care in fractured and inefficient ways. The data analyzed here serve as a preliminary understanding of the target population and begin to illustrate the areas for improvement. The Comprehensive Care Program for Sickle Cell Disease at the Center for Special Health Care Needs is the first step in addressing this patient population. Primary care services are provided by four primary care physicians, all trained in both Internal Medicine and Pediatrics, as well as advanced practitioner support from a nurse practitioner and a physician assistant. There is specialized hematology/oncology expertise. Additionally, an adult hematologist is on-site during the clinic times for further consultations. Patients with sickle cell disease have a dedicated social worker, and the Center as a whole has a case manager. Patients will be tagged in the EHR, allowing real-time notification of the team when they are admitted or checked into an ED, allowing improved coordination and personalization of care. Targeted and focused interventions for this cohort of adolescent/young adult patients with sickle cell disease have the potential to improve quality of life, decrease the cost of care, and improve quality of care.

## Figures and Tables

**Figure 1:**
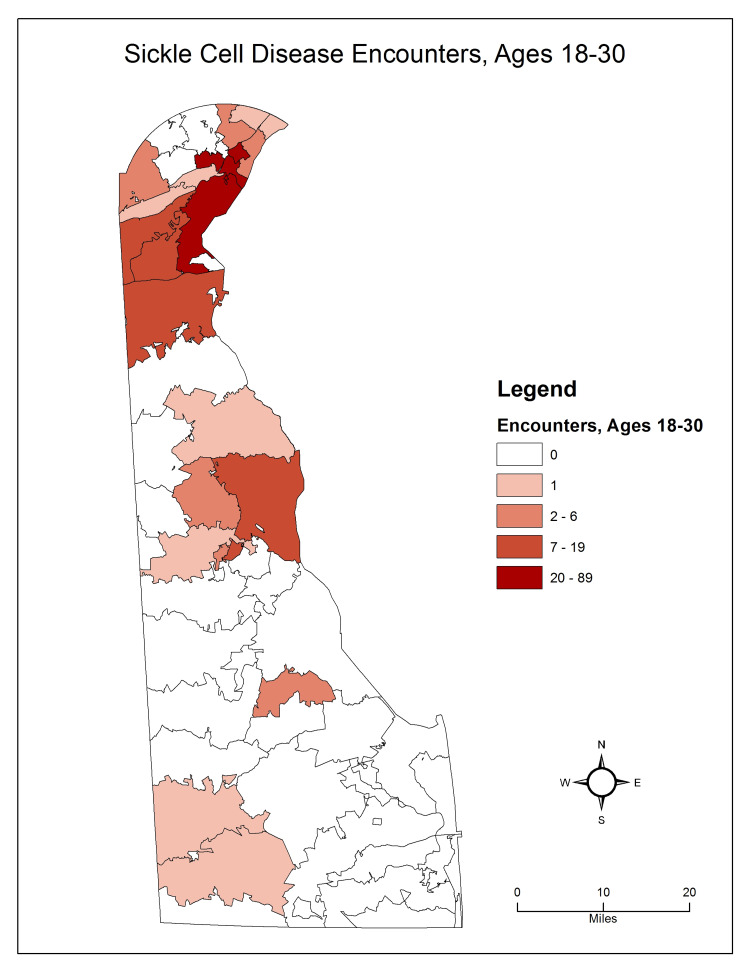
Sickle Cell Disease Encounters, Age 18-30

## Appendices

Appendix 1

## References

[1] (2022). Centers for Disease Control and Prevention: Data & statistics on sickle cell disease. https://www.cdc.gov/ncbddd/sicklecell/data.html.

[2] Yanni E, Grosse SD, Yang Q, Olney RS (2009). Trends in pediatric sickle cell disease-related mortality in the United States, 1983-2002. J Pediatr.

[3] Hamideh D, Alvarez O (2013). Sickle cell disease related mortality in the United States (1999-2009). Pediatr Blood Can.

[4] Lanzkron S, Carroll CP, Haywood C Jr (2013). Mortality rates and age at death from sickle cell disease: U.S., 1979-2005. Public Health Rep.

[5] Bou-Maroun LM, Meta F, Hanba CJ, Campbell AD, Yanik GA (2018). An analysis of inpatient pediatric sickle cell disease: Incidence, costs, and outcomes. Pediatr Blood Can.

[6] Monaghan M, Baumann K (2016). Type 1 diabetes: addressing the transition from pediatric to adult-oriented health care. Res Rep Endocr Disord.

[7] Kayle M, Shah N, Tanabe P, Sloane R, Maslow G, Pan W, & Docherty S (2017). Health care utilization trajectories and associated factors for transitioning adolescents/young adults with sickle cell disease. Blood.

[8] Treadwell M, Telfair J, Gibson RW, Johnson S, Osunkwo I (2011). Transition from pediatric to adult care in sickle cell disease: establishing evidence-based practice and directions for research. Am J Hematol.

[9] Raphael JL, Rattler TL, Kowalkowski MA, Brousseau DC, Mueller BU, Giordano TP (2013). Association of care in a medical home and health care utilization among children with sickle cell disease. J Natl Med Assoc.

[10] Gardner K, Douiri A, Drasar E, Allman M, Mwirigi A, Awogbade M, Thein SL (2016). Survival in adults with sickle cell disease in a high-income setting. Blood.

[11] Blinder MA, Duh MS, Sasane M, Trahey A, Paley C, Vekeman F (2015). Age-related emergency department reliance in patients with sickle cell disease. J Emerg Med.

[12] Adams-Graves P, Bronte-Jordan L (2016). Recent treatment guidelines for managing adult patients with sickle cell disease: challenges in access to care, social issues, and adherence. Expert Rev Hematol.

[13] Sandhu MK, Cohen A (2015). Aging in sickle cell disease: Co-morbidities and new issues in management. Hemoglobin.

[14] Whiteman LN, Haywood C Jr, Lanzkron S, Strouse JJ, Feldman L, Stewart RW (2015). Primary care providers' comfort levels in caring for patients with sickle cell disease. South Med J.

[15] Reeves S, Garcia E, Kleyn M, Housey M, Stottlemyer R, Lyon-Callo S, Dombkowski KJ (2014). Identifying sickle cell disease cases using administrative claims. Acad Pediatr.

[16] Lanzkron S, Carroll CP, Hill P, David M, Paul N, Haywood C Jr (2015). Impact of a dedicated infusion clinic for acute management of adults with sickle cell pain crisis. Am J Hematol.

